# Determinants of self-management behaviors among pulmonary tuberculosis patients: a path analysis

**DOI:** 10.1186/s40249-021-00888-3

**Published:** 2021-07-30

**Authors:** Jin Li, Jie Pu, Jiaqing Liu, Qingya Wang, Rui Zhang, Ting Zhang, Jiani Zhou, Wei Xing, Shengxiang Liang, Daiyu Hu, Ying Li

**Affiliations:** 1 Department of Social Medicine and Health Service Management, Army Medical University (Third Military Medical University), Chongqing, China; 2 Chongqing Institute of TB Prevention and Treatment, Jiulongpo District, Chongqing, China

**Keywords:** Self-management, Tuberculosis, PRECEDE, Path analysis

## Abstract

**Background:**

Tuberculosis (TB) is one of the top 10 causes of death in the world. Since Directly Observed Therapy (DOT) as a core strategy for the global TB control are not applicable to all types of TB patients, and self-management of TB patients (SMTP) as a patient-centered supervision type is a supplement to DOT and can improve TB case management. However, the factors related to SMTP are complex and need more study. This study aimed at identifying the determinants of SMTP and examining the direct/indirect effects of these determinants.

**Methods:**

The purposive sampling technique was used to select study sites and participants were recruited from the study sites by the consecutive sampling method. The PRECEDE model was used as the framework to analyze the determinants of SMTP. The responses of TB patients were acquired via a questionnaire survey for data collection. A Pearson correlation analysis was used to define the relationship between the predisposing, enabling, reinforcing factors with SMTP behaviors. A regression-based path analysis was used to determine the action paths of the predisposing, enabling, and reinforcing factors on SMTP behaviors.

**Results:**

The predisposing (TB knowledge), enabling [health education and healthcare workers (HCWs) support], reinforcing factors (family support) had significant positive correlations with SMTP behaviors (*P* < 0.05). The predisposing, enabling, reinforcing factors were positively correlated with each other (r = 0.123‒0.918, *P* < 0.05), except for family support and HCWs support. The predisposing factors (TB knowledge, β = 0.330) and the enabling factors (HCWs support, β = 0.437) had direct effects on SMTP behaviors. The enabling factors (health education and HCWs support) and the reinforcing factors (family support) had indirect effects on SMTP behaviors.

**Conclusions:**

This study revealed the effects and action path of TB knowledge, health education, HCWs support, and family support on SMTP behaviors via a path analysis. Assessing patient’s needs for SMTP along with promoting effective TB health education and providing firm support from HCWs and family members are potential strategies to promote SMTP behaviors.

**Graphic abstract:**

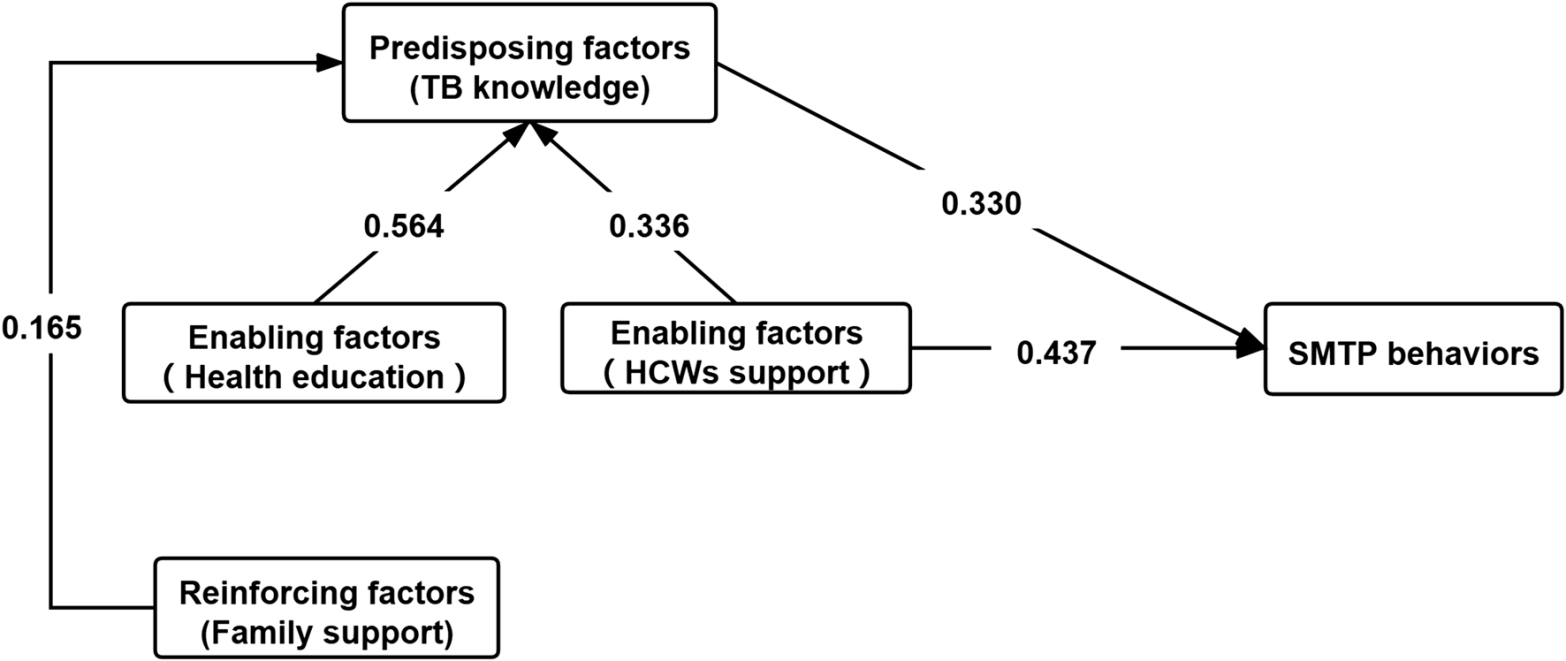

## Background

Tuberculosis (TB) is caused by the bacillus *Mycobacterium tuberculosis*, is one of the most ancient infectious diseases of mankind. It is the most deadly infections disease and one of the top 10 leading causes of death [1]. TB can affect anyone, anywhere. In 2019, there were an estimated 10.0 million new cases of TB and 1.4 million TB-caused deaths worldwide [1]. India, Indonesia, and China were the three countries with the highest TB burden. Drug-resistant TB worsens the progress of TB control and is a public health threat. Though the global commitments and strategies in the fight against TB were intensified, the number of TB cases worldwide has still been declining very slowly in recent years [1].

One of the key TB control policies recommended by the World Health Organization (WHO) in 1992 is directly observed therapy short course (DOTS) [2]. Directly observed therapy (DOT) as one of the key components of DOTS is implemented when patients are required to swallow their medications under direct observation, which is necessary in order to decrease poor treatment adherence among TB patients due to a long treatment duration lasting at least 6 months required for TB [3]. DOTS coverage has reached 100% in China [4, 5] and the WHO has announced China’s National Tuberculosis Control Program that uses the DOTS strategy “One of the most successful DOTS-programs in the world [6]”.However, DOT did not achieve better results as expected [7, 8]. A meta-analysis [9] on the implementation of the DOTS strategy in China stated that the proportion of TB patients whose treatment was strictly observed was much lower than that reported by official statistics in China, only 20% TB patients were monitored by health workers. Our previous study [10] also observed that more than 1/3 of TB patients in Chongqing were never monitored by any healthcare workers (HCWs). In addition, DOT cannot truly support all types of patients for a variety of reasons, including shortage of primary care medical staff, inconvenient transportation, and patient concerns regarding privacy [11, 12]. Therefore, it is necessary that new TB management strategies as effective supplements to DOT be explored.

The Stop TB Strategy in 2014 recommended that patient-centered care and supervision must be carried out in a context-specific and patient-sensitive manner [13]. Self-management could be a patient-centered way that can be employed for patients that are unwilling to accept DOT or with poor accessibility to DOT. Self-management as a new type of disease intervention requires that the individual patient (and his or her family) are actively engaged and willing to participate in the treatment of his/her own illness [14], which is often successfully applied for the case management of chronic diseases (hypertension, diabetes, and so on). For example, previous studies on self-management of hypertension among diabetes patients confirmed that self-management could help improve the patient’s condition and establish health behaviors, therefore improving the patient’s quality of life [15–17] and promoting medication adherence and good treatment outcomes [18, 19]. In China, there are some research studies on the self-management of TB patients (SMTP), some of which have explored tools to measure SMTP [20, 21], and some studies reported promoting SMTP through health education [22, 23]. Individual studies evaluated the impact of SMTP on TB treatment and found that it promoted good treatment outcomes [24, 25]. Why SMTP could do so? SMTP as a type of patient-centered supervision needs to identify and address factors that may prevent patients from treatment interruption [13]. However, few studies have explored the factors related to SMTP. Although there are individual reports examining the effects of demographic factors as well as the disease characteristics of SMTP [26–28] showing that gender, age [26], education level, work status and economic income, the duration of diagnosis, frequency of hospitalization [27], and drug side effects [28] are associated with SMTP in China.

Any behavior is associated with many factors, including individual self-factors (biological and psychological factors), inter-individual factors (social and cultural factors), and environmental factors (natural and social environment factors) [29]. However, current research on the factors related SMTP only focused on individual factors, and so it is necessary to systematically study the determinants of SMTP, which would provide evidence with which to build SMTP interventions. According to the Predisposing, Reinforcing, and Enabling Constructs in Educational/Environmental Diagnosis and Evaluation (PRECEDE) model, we divide these factors into the predisposing, enabling, and reinforcing factors.

This study aimed at surveying the determinants of SMTP using the PRECEDE model, analyzing the direct and indirect effects of these, and determining the pathway of SMTP via a pathway analysis.

## Methods

### Study setting

We purposively selected the Chongqing municipality [a region with a relatively developed socio-economic status, with a gross domestic product (GDP) of 2.04 trillion CNY and a per capita GDP at 66.2 thousand CNY] and the Guizhou Province (a region with relatively less developed socio-economic conditions, with a GDP of 1.48 trillion CNY and a per capita GDP of 41.4 thousand CNY) as the study regions [30]. The incidence of tuberculosis in the Guizhou Province is ranked third in China (133.5/100 000), and the incidence in Chongqing City is ranked tenth (75.0/100 000), both of which are a part of the provinces with high TB epidemic rates in China [31]. More than one third of TB patients in Chongqing and 62.9% of TB patients in Guizhou [10, 12] do not swallow their medications under direct observation during their entire treatment period. According to the ranking of the TB epidemic situation in the districts and counties of Chongqing and Guizhou in 2017, with specific factors such as geographical location and economic status comprehensively considered, a total of 12 districts/counties were selected as the study sites for this study, and 71 Primary Health Care sectors, including community health service centers (CHCs) and township hospital centers (THCs) in selected districts/counties were included in this study.

### Sample size determination

Sample size was estimated using Kish and Leslie formula as follows [32]:

$${{n}} = {\text{Z}}\alpha^{{2}} {\text{p}}\left( {{1 } - {\text{p}}} \right)/{\text{d}}^{{2}}$$

where: *n* = Minimum desired sample size. Zα = the standard normal deviate, usually set as 1.96 which corresponds to 5% level of significance. *P* = the average rate of SMTP was estimated on the basis of the available literature [33, 34], and its value was set at 59.5%. d = Degree of accuracy (precision) set at 5% (0.05).

The calculated minimum sample size was 370 (*n* = 1.96^2^ × 0.595 × (1 − 0.595)/0.05^2^ = 370).

### Framework of determinants of SMTP

**Fig. 1:**
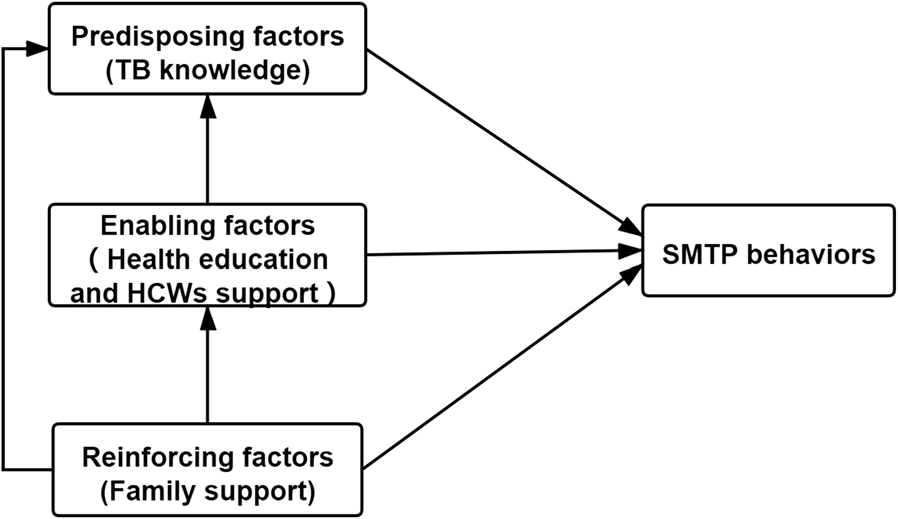
The hypothetic relationship between the predisposing, enabling, reinforcing factors and SMTP behaviors. This figure presents the hypothetic relationship between the predisposing, enabling, reinforcing factors and SMTP behaviors. The predisposing, reinforcing, enabling factors each have a direct predictive effect on SMTP behaviors. The enabling factors can indirectly predict behaviors through the predisposing factors. The reinforcing factors can act indirectly on SMTP behaviors through the predisposing factors and the enabling factors, respectively. HCWs refer to healthcare workers; SMTP refer to self-management of tuberculosis patients

The PRECEDE-PROCEED model developed by Green and Kreuter is an ecological approach to health promotion [35]. PRECEDE stands for Predisposing, Reinforcing, and Enabling Constructs in Educational/Environmental Diagnosis and Evaluation, which are three kinds of factors associated with behaviors [36]. The predisposing factors are related to the internal basis for the occurrence of a behavior and are subjective factors, such as knowledge, attitude, motivation, and so on; the enabling factors are objective factors that enable the realization of predisposing factors, such as skills, resources, and social conditions; and the reinforcing factors refer to the incentives that encourage one to continually perform a behavior [29]. In this study, we used the PRECEDE model as our framework. The TB knowledge of pulmonary tuberculosis (PTB) patients was selected as the predisposing factors, the health education and support from HCWs that was received by PTB patients was selected as the enabling factors, and the family support received by PTB patients was selected as the reinforcing factors. Based on the PRECEDE model, this study makes the following assumptions:

1. The predisposing, reinforcing, and enabling factors have a direct predictive effect on SMTP behaviors;
2. The enabling factors can indirectly predict SMTP behaviors through the predisposing factors;
3. The reinforcing factors can act indirectly on SMTP behaviors through the predisposing factors and enabling factors, respectively (Fig. 1).

### Study participants

This study used the consecutive sampling method to recruit participants. During our study period, all PTB patients that were admitted to the 71 THCs/CHCs that met the following inclusion criteria were recruited as participants: (1) they were registered at TB dispensaries and diagnosed with PTB according to the WHO guidelines; (2) they received close to 6 months’ standard TB treatment or finished a standard TB treatment in the past 4 months; and (3) were at least 15 years of old. The exclusion criteria included: (1) patients with extra-pulmonary TB; (2) patients with speech or hearing difficulties; and (3) patients who declined to participate. Participants were recruited through the Center for Disease Control of the selected counties/districts. All PTB patients who were interested in our study and met the inclusion criteria were approached and were asked for their informed consent.

### Data collection and measures

A structured questionnaire survey was conducted by trained investigators in clinic rooms within the THCs/CHCs after pre-test. The following information are covered in the survey:

1. Socio-demographic information: age, sex, occupation, ethnicity, education, registered residence, marital status, and so on.
2. TB knowledge: TB knowledge was measured using six questions, including transmission routes, symptoms, consequences of irregular treatment, TB treatment related policies, treatment institutions, and whether it could be cured. One point was allotted for each correct answer, totaling six points.
3. Health education: health education was assessed through nine items of health education provided by healthcare workers, including the importance of regular treatment, course of treatment, taking and storing drugs, side effects, regular follow-up sputum exams, lifestyle guidance, adherence to medications, the national TB treatment policy, contact screening. One point was allotted for each education item received, totaling nine points.
4. HCWs support from primary health care sections: we assessed HCWs support through whether the respondents received HCWs support at different treatment stages (TB intensive treatment and TB continuous treatment). The HCWs support was defined as the workers reminding the patients to take their medicine and performing the follow-up sputum exams during the treatments. Receiving one of reminders by a HCWs counted as one point, for a total of two points.
5. Family support: the family support included economic support, nutritional support, and psychological support, and one point was scored for each type of family support received, for a total of three points.
6. SMTP: SMTP behavior scores were assessed using five questions regarding the five SMTP items: managing missing doses, interrupted treatments, regular follow-up sputum exams, and side effect and contact screening, all of which are defined according to the Treatment Management for Tuberculosis Patients in the Technical Specifications for Tuberculosis Prevention and Control in China (2020 Edition) [37]. One correct answer was counted as one point, totaling five points.

### Quality control

Before the formal survey, the questionnaire was modified based on the results of the pre-survey. The investigators were trained uniformly to ensure they mastered the survey skills; and the purpose and significance of the survey were explained to participants with uniform guidance. After survey, all questionnaires were checked everyday by research team. Besides, 10% of the questionnaires were randomly selected and re-reviewed everyday, if more than 5% of those questionnaires data cannot be repeated, they would be finally excluded in final analysis and retrain the investigators.

### Data analysis

We adopted EpiData 3.0 (The EpiData Association, Odense, Denmark) for data entering and the Statistical Package for Social Science (SPSS 19.0, IBM Corporation, Armonk, NY, USA) for data analysis. A probability level of *P* < 0.05 was considered statistically significant. A Pearson correlation analysis was used to define the relationship between the predisposing, enabling, and reinforcing factors with SMTP behaviors.

A regression-based path analysis was used to determine the action paths of the predisposing, enabling, and reinforcing factors on SMTP behaviors. Since the variables involved were all distributed in a skewed manner, a generalized linear regression was chosen for all regression analyses. The specific steps of the path analysis were as follows:

Step 1: Regression analyses were performed with the SMTP behavior scores as the dependent variables and the predisposing factors (TB knowledge), enabling factors (health education and HCWs support), and reinforcing factors (family support) as the independent variables to assess the predictive ability of the three factors for SMTP behaviors.

Step 2 to 4: Examined the mediating role of the predisposing factors (TB knowledge) on the enabling factors (health education) in SMTP behaviors. In Step 2: a regression analysis was performed with the enabling factors (health education) as independent variables and the predisposing factors (TB knowledge) as dependent variables. In step 3: a regression analysis was performed with the enabling factors (health education) and the predisposing factors (TB knowledge) as independent variables and SMTP behaviors as dependent variables to investigate the common effect of the two independent variables on SMTP behaviors. In step 4: a regression analysis was performed to examine the total effects from the enabling factors (health education) on SMTP behaviors.

Step 5 to 7: Examined the mediating role of the predisposing factors (TB knowledge) on the enabling factors (HCWs support) in SMTP behaviors. In step 5: a regression analysis was performed with the enabling factors (HCWs support) as independent variables and the predisposing factors as dependent variables. In step 6: the enabling factors (HCWs support) and the predisposing factors (TB knowledge) were entered into the regression equation as independent variables to test their effects on SMTP behaviors. In step 7: a regression analysis was performed to evaluate the total effects from the enabling factors (HCWs support) on SMTP behaviors.

Step 8 to 10: Explored the mediating role of the predisposing factors (TB knowledge) on the reinforcing factors (family support) for SMTP behaviors. In step 8: a regression analysis was performed with the reinforcing factors as independent variables and the predisposing factors as dependent variables. In step 9: the reinforcing factors (family support) and the predisposing factors (TB knowledge) were entered into the regression equation as independent variables to test their effects on SMTP behaviors. In step 10: a regression analysis was performed to test the total effects from the reinforcing factors (family support) on SMTP behaviors.

Step 11 examined the mediating role of the enabling factors (health education) on the reinforcing factors (family support) in terms of SMTP behaviors. A regression analysis was performed with the reinforcing factors as independent variables and the enabling factors (health education) as dependent variables.

During the process, univariate and multivariate analyses were performed for the five variables involved in the predisposing, enabling, and reinforcing factors, as well as SMTP behaviors, respectively, in order to determine the factors related to each variable. According to the different independent and dependent variables included in each regression, the common variables were included as control variables in the regression analysis to ensure the true reflection of the relationship between the predisposing, enabling, reinforcing factors and SMTP behaviors.

The standardized beta values of the generalized linear regression analysis from the first step to the eleventh step were considered as the path coefficients, which are estimations of the direct effect of the independent variables on the dependent variable. To determine the indirect effects of independent variables on the dependent variable, the beta values of the indirect paths were multiplied by each other. The total effect of the independent variables on the dependent variable was calculated by adding the total multiples of the direct and indirect pathways.

## Results

### Demographic characteristics of PTB patients

A total of 465 patients completed the questionnaire survey and 459 were included in the final analysis after the exclusion of missing data. Most participants were male patients (70%, 324) and aged 40 and above (79.5%, 365). A high proportion of the patients (82.4%, 378) were rural residents. Close to 70% (*n* = 320) of the patients were married. More than half (56.0%, *n* = 257) of the patients had primary school and below education backgrounds, and more than two thirds (*n* = 315) of the patients were farmers or migrant workers. Almost all of the patients (96.7%, *n* = 444) had basic medical insurance. More than 40% (*n* = 193) of the patients reported that their main source of income was from family support (Table 1).

**Table 1:** Demographic characteristics of pulmonary tuberculosis patients in questionnaire survey (n = 459)

Demographic characteristics	Frequency	Percentage
Gender		
Male	324	70.6
Female	135	29.4
Age		
< 20	18	3.9
20–39	76	16.6
40–59	174	37.9
≥ 60	191	41.6
Ethnicity		
Han Race	359	78.2
Others	100	21.8
Residence		
Urban	81	17.6
Rural	378	82.4
Registered information		
Resident	437	95.2
Migrant	22	4.8
Marital status		
Unmarried	69	15.0
Married	320	69.7
Divorced/Widowed	70	15.3
Education		
Primary and below	257	56.0
Junior middle school	125	27.2
High school and above	77	16.8
Occupation		
Staff/Cadre/Retire	50	10.9
Self-employed	10	2.2
Farmer/Migrant worker	315	68.6
Student	20	4.4
Others	64	13.9
Work/studying status		
Working/studying	108	23.5
Frequently on leave	51	11.1
Stop working/studying completely	221	48.2
No working/studying	79	17.2
Health insurance		
Basic medical insurance	444	96.7
Non Basic medical insurance	6	1.3
No medical insurance	9	2.0
Main source of income		
Fixed income	40	8.7
Self-employed	4	0.9
No Fixed income	150	32.7
Family support	193	42.0
Insurance	18	3.9
Subsistence allowances\relief	19	4.2
Others	35	7.6

### Correlation between the predisposing, enabling, reinforcing factors and SMTP behaviors

The results of the Pearson correlation analysis showed statistically significant positive correlations between the predisposing, enabling, reinforcing factors and SMTP behaviors (*P* < 0.05). The correlation coefficient varied from 0.114 (for the relationship between family support and SMTP behaviors) to 0.301 (for the relationship between TB knowledge and SMTP behaviors). The predisposing, enabling, reinforcing factors were positively correlated with each other (r = 0.123‒0.918, *P* < 0.05), except for family support and HCWs support (r = 0.093, *P* ˃ 0.05) (Table 2).

**Table 2:** Pearson Correlation Coefficients between the predisposing, enabling, reinforcing factors and SMTP behaviors (r)

	Predisposing factors (TB knowledge)	Enabling factors (health education)	Enabling factors (HCWs support)	Reinforcing factors (family support)	SMTP behaviors
Predisposing factors (TB knowledge)	1				
Enabling factors (health education)	0.918**	1			
Enabling factors (HCWs support)	0.292**	0.300**	1		
Reinforcing factors (family support)	0.145**	0.123*	0.093	1	
SMTP behaviors	0.301**	0.280**	0.215**	0.114*	1

HCWs: Healthcare workers, SMTP: Self-management of tuberculosis patients

r represents Pearson Correlation Coefficient

*P < 0.05, **P< 0.01

### Regression-based path analysis of the predisposing, enabling, reinforcing factors constructs to predict SMTP behaviors

The variables (TB knowledge, health education, HCWs support, family support) that had statistically significant correlations with SMTP behaviors in the Pearson correlation analysis were entered to explore their pathways in terms of SMTP behaviors (Table 3).

**Table 3:** Regression-based path analysis of the predisposing, enabling, reinforcing factors constructs to predict SMTP behaviors

STEP	Independent variable	Standardized Beta	*P value*	Dependent variable
1^a^	Predisposing factors (TB knowledge)	0.330	0.003	SMTP behaviors
	Enabling factors (health education)	-0.065	0.360	
	Enabling factors (HCWs support)	0.437	0.003	
	Reinforcing factors (family support)	0.085	0.093	
2^b^	Enabling factors (health education)	0.564	0.000	Predisposing factors (TB knowledge)
3^c^	Enabling factors (health education)	0.009	0.531	SMTP behaviors
	Predisposing factors (TB knowledge)	0.263	0.000	
4^d^	Enabling factors (health education)	0.152	0.000	SMTP behaviors
5^e^	Enabling factors (HCWs support)	0.366	0.000	Predisposing factors (TB knowledge)
6^f^	Enabling factors (HCWs support)	0.134	0.021	SMTP behaviors
	Predisposing factors (TB knowledge)	0.246	0.000	
7^g^	Enabling factors (HCWs support)	0.246	0.000	SMTP behaviors
8^h^	Reinforcing factors (family support)	0.165	0.003	Predisposing factors (TB knowledge)
9^i^	Reinforcing factors (family support)	0.098	0.056	SMTP behaviors
	Predisposing factors (TB knowledge)	0.270	0.000	
10^j^	Reinforcing factors (family support)	0.118	0.027	SMTP behaviors
11^k^	Reinforcing factors (family support)	0.164	0.117	Enabling factors (health education)

HCWs: Healthcare workers, SMTP: Self-management of tuberculosis patients, TB: Tuberculosis

^a^Occupation as control variables;

^b^Age, marital status, education, occupation, main source of income as control variables;

^c^Age, marital status, occupation as control variables;

^d^Age, marital status, occupation as control variables;

^e^Marital status, education, occupation, main source of income as control variables;

^f^Marital status, occupation as control variables;

^g^Marital status, occupation as control variables;

^h^Age, occupation, main source of income as control variables;

^i^Age, occupation as control variables;

^j^Age, occupation as control variables;

^k^Age, occupation, work/studying status, main source of income as control variables

Multiple regression of the predisposing, enabling, reinforcing factors on SMTP behaviors (Table 3, step 1): step 1 of the generalized linear regression was conducted to assess the abilities of the predisposing, enabling, reinforcing factors in predicting SMTP behaviors. The results showed that the predisposing factors (TB knowledge) and the enabling factors (HCWs support) positively predicted SMTP behaviors (TB knowledge, β = 0.330; HCWs support, β = 0.437).

The mediating role of the predisposing factors (TB knowledge) on the enabling factors (health education) in terms of SMTP behaviors (Table 3, steps 2‒4): At step 2, the enabling factors (health education) had positive predictive effects on the predisposing factors (TB knowledge, β = 0.564).At step 3 when the enabling factors (health education) and the predisposing factors (TB knowledge) were entered together into the regression equation, the enabling factors (health education) could not significantly predict SMTP behaviors, while the predisposing factors (TB knowledge) had positively predictive effects on SMTP behaviors (β = 0.263). At step 4, the total effect of the enabling factors (health education) in the prediction of SMTP behaviors was significant (β = 0.152). The above results demonstrated that the enabling factors (health education) had no direct effects on SMTP behaviors and can have indirect effects on SMTP behaviors through the predisposing factors (TB knowledge).

The mediating role of the predisposing factors (TB knowledge) on the enabling factors (HCWs support) in terms of SMTP behaviors (Table 3, steps 5‒7): At step 5, the enabling factors (HCWs support) had positive predictive effects on the predisposing factors (TB knowledge, β = 0.366).At step 6 when the enabling factors (HCWs support) and the predisposing factors (TB knowledge) were entered together into the regression equation, both the enabling factors (HCWs support, β = 0.134) and the predisposing factors (TB knowledge, β = 0.246) positively predicted SMTP behaviors. At step 7, the total effect of the enabling factors (HCWs support) in the prediction of SMTP behaviors was significant (β = 0.246). The above results demonstrated that the enabling factors (HCWs support) may affect SMTP behaviors directly and that they have indirect effects on SMTP behaviors through the predisposing factors (TB knowledge).

The mediating role of the predisposing factors (TB knowledge) on the reinforcing factors (family support) in terms of SMTP behaviors (Table 3, steps 8‒10): At step 8, the reinforcing factors (family support) had positive predictive effects on the predisposing factors (TB knowledge, β = 0.165). At step 9 when the reinforcing (family support) and the predisposing factors (TB knowledge) were entered together into the regression equation, the reinforcing factors (family support) could not significantly predict SMTP behaviors, while the predisposing factors (TB knowledge) had positive predictive effects on SMTP behaviors (β = 0.270). At step 10, the total effect of the reinforcing factors (family support) in the prediction of SMTP behaviors was significant (β = 0.118). These results indicated that the reinforcing factors (family support) have no direct effect on SMTP behaviors and can have indirect effects on SMTP behaviors through the predisposing factors (TB knowledge).

The mediating role of the enabling factors (health education) on the reinforcing factors (family support) in terms of SMTP behaviors (Table 3, step 11): At step 11, the reinforcing factors (family support) could not significantly predict the enabling factors (health education) (β = 0.164, *P* > 0.05). Therefore, the reinforcing factors (family support) did not have indirect effects on SMTP behaviors through the enabling factors (health education).

### Direct, indirect, and total effects of independent predictors on SMTP behaviors

**Fig. 2:**
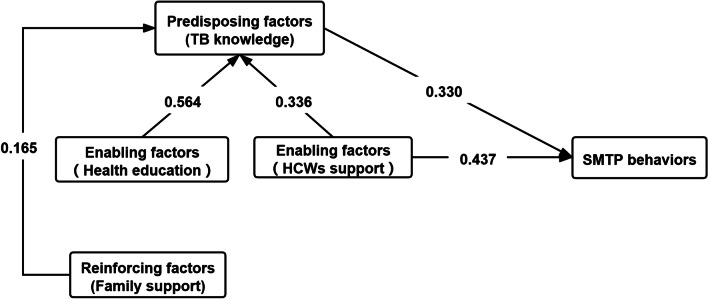
Regression-based path analysis of the predisposing, enabling, and reinforcing factors on SMTP behaviors. This figure presents the regression-based path analysis of the predisposing, enabling, reinforcing factors on SMTP behaviors in the present study. The predisposing factors (TB knowledge) and the enabling factors (HCWs support) have direct effects on SMTP behaviors. The enabling factors (health education and HCWs support) and the reinforcing factors (family support) have indirect impact on SMTP behaviors through the predisposing factors (TB knowledge). HCWs refer to healthcare workers; SMTP refer to self-management of tuberculosis patients

The pathway impact of the predisposing, enabling, reinforcing factors on SMTP behaviors were presented in Fig. 2.The predisposing factors (TB knowledge) only had direct impacts on SMTP behaviors, while the enabling factors (health education, β = 0.564) and reinforcing factors (family support, β = 0.165) only had indirect impacts on SMTP behaviors via the predisposing factors (TB knowledge). The enabling factors (HCWs support, β = 0.437) may affect SMTP behaviors directly, and it also had indirect effects on behaviors through the predisposing factors (TB knowledge, β = 0.336).Values for the direct, indirect, and total effects of independent predictors on SMTP behaviors are described in Table 4. The enabling factors (HCWs support) had both direct impacts (β = 0.437) and indirect impacts (0.054) on SMTP, and the total effect (0.558) was maximal. The reinforcing factors (family support) had only indirect effects (0.054) on SMTP behaviors, and the total effect was minimal.

**Table 4:** Direct, indirect and total effects of independent predictors on SMTP behaviors based on regression-based path analysis

Independent variables	Direct effect*	Indirect effect**	Total effect***	Dependent variables
Predisposing factors (TB knowledge)	0.330	-	0.330	SMTP behaviors
Enabling factors (health education)	-	0.564 × 0.330 = 0.186 (effect of health education on TB knowledge × effect of TB knowledge on SMTP behaviors)	0.186	
Enabling factors (HCWs support)	0.437	0.366 × 0.330 = 0.121 (effect of HCWs support on TB knowledge × effect of TB knowledge on SMTP behaviors)	0.558	
Reinforcing factors (family support)	-	0.165 × 0.330 = 0.054 (effect of family support on TB knowledge × effect of TB knowledge on SMTP behaviors)	0.054	

HCWs: Healthcare workers, SMTP: Self-management of tuberculosis patients

*The direct effect means that the independent variable has direct effect on SMTP behaviors

**The indirect effect means that the independent variable has indirect effect on SMTP behaviors through other variables

***The total effect is the sum of direct and indirect effects of independent variable on SMTP behaviors

## Discussion

DOT cannot cover all kind of TB patients anywhere [38, 39]. Evidence has indicated that STMP as a patient-centered disease management strategy can effectively promote patient adherence to TB treatment [40, 41]. This study identified the determinants of STMP for treatment adherence including the predisposing factors (TB knowledge), enabling factors (health education and medical staff support), and reinforcing factors (social support).

The predisposing factors (knowledge), as the intrinsic basis for the occurrence of behaviors, are the original drivers of the occurrence of behaviors [29]. A previous study reported that enhanced knowledge is important for improving self-management behaviors among osteoporosis patients [42]. Knowledge can change patients’ cognition, help improve patients’ self-efficacy, and establish good self-management behaviors [42]. Indeed, to self-manage themselves, patients must have knowledge and resources to deal with illness-related issues as they arise [43]. The regression-based path analysis showed that the predisposing factors (TB knowledge) were the best predictors of SMTP behaviors, and the enabling and the reinforcing factors had an indirect impact on SMTP behaviors through the predisposing factors (TB knowledge).These results indicate that more attention should be paid to the promotion of TB patients' knowledge in order to improve their cognition and understanding of TB. Furthermore, several studies suggest that the sense and perceptions of health-related risk appear to play a more important role in motivating behavior changes, and patients’ fear of hazards can lead to the development of motivation and increase their commitment to adopting new behaviors [44, 45]. Hence, the risk and hazards due to patients’ noncompliance with the standard treatment should be emphasized to TB patients through knowledge improvement, which may trigger their changes of behavioral motivation and actively lead to them adopting adherence behaviors.

Education was found to be effective in increasing awareness [46]. Health education as a stimulus, or cue to action, might trigger behaviors related improving one’s health [47]. This would explain the results from the path analysis in this study, where the enabling factors (health education) did not have a direct impact on SMTP behaviors, but health education as an enabling factor had an indirect impact on SMTP behaviors through increases in TB knowledge. This suggests that TB health education would have an impact on SMTP behaviors if TB health education could be translated into TB knowledge and awareness. Furthermore, a randomized controlled trial in Ethiopia found that educational interventions significantly decreased treatment non-adherence among TB patients [48]. Since 2008, the Guidelines on Enforcement of Chinese Tuberculosis Control Program [49] emphasized the importance of TB health education/health promotion in China. However, a previous study found that less than 70% of the TB patients in China had TB knowledge [50]. Therefore, further research is required on the strategies that can improve effects from TB health education programs on TB health knowledge among Chinese TB patients.

It has been reported that supportive interventions by health care staff can promote self-management behaviors [42, 51]. Similarly, this study observed that HCWs support (reminding TB patients to take their medications and follow-up exams) from PHC sections as an enabling factor had a direct impact on patients’ SMTP behaviors. A previous study on the effects of reminders through the sending of text messages by medical staff on TB patient adherence concluded that reminder messages should target to different stages of TB treatment and to the psychological needs of the TB patients, and also motivate, inform, and facilitate patients to overcome all obstacles during treatment [52]. Therefore, before HCWs from PHC sections can prepare and provide corresponding support to TB patient and facilitate SMTP behaviors, the needs of TB patient that are related to supportive interventions should be carefully assessed.

A meta-analysis demonstrated that family plays a crucial role in effective patient self-management, which can contribute to better health outcomes for chronic diseases [53]. TB as a chronic infectious disease has a long treatment period and several side effects [54], which often impede TB patient adherence to treatment regimens [55, 56]. Previous study found that family members also play a role in the supervision of PTB treatment, and family support as an emotional support can improve patient’s confidence and promote their adherence to treatment [57]. This study found that family support as a reinforcing factors also had indirect effects on SMTP behaviors through the predisposing factors (TB knowledge). Family support might influence an individual's self-care behaviors by enhancing motivation, providing information, and giving feedback [58]. Enhancing family support for TB patients may encourage TB patients to maintain SMTP behaviors persistently by receiving economic, nutritional, and psychological support by family members.

### Strengths and limitations

It has been reported in previous studies that focused on TB knowledge and health education concluded that these have an important impact on TB patient adherence to treatment [59], and DOT provided by HCWs through text messages and telephone calls can effectively improve TB patient’s adherence to anti-TB treatments [52], which shows that previous studies focused on the impact of TB knowledge, health education, DOT, and family support on TB treatment adherence or TB treatment outcomes. In contrast, this study tried to explore the relationship between the above factors on SMTP behaviors and figure out how the above factors predict SMTP behaviors via a path analysis under the guidance of the PRECEDE model. Results of this study provided primary evidence for the development of more effective interventions to promote SMTP and TB control.

This study has some limitations. Firstly, the predisposing factors involved in this study is limited to the patient’s TB knowledge; patient attitudes towards TB and self-efficacy need to be included as predisposing factors in order to predict SMTP in future studies. Secondly, the reinforcing factors of SMTP only included family support, peer support from other TB patients should be included in subsequent studies in order to predict SMTP behaviors.

## Conclusions

This study revealed the effects and action path of the predisposing (TB knowledge), enabling (health education and HCWs support), reinforcing factors (family support) on SMTP behaviors through a path analysis based on the PRECEDE model. SMTP as a type of patient-centered TB management strategy could become one necessary supplement to DOT. Assessing patient’s needs for SMTP, promoting effective TB health education, and providing corresponding supports from HCWs and family are possible effective strategies to promote SMTP.

## Data Availability

The datasets used and/or analysed during the current study are available from the corresponding author on reasonable request.

## Abbreviations

CHCs: Community health center
DOT: Directly observed therapy
DOTS: Directly observed therapy, short-course
GDP: Gross domestic product
HCWs: Healthcare workers
PTB: Pulmonary tuberculosis
SMTP: Self-management of tuberculosis patients
TB: Tuberculosis
THCs: Township hospital center
WHO: World Health Organization
